# Ion Channel Function During Oocyte Maturation and Fertilization

**DOI:** 10.3389/fcell.2018.00063

**Published:** 2018-06-26

**Authors:** Ingrid Carvacho, Matthias Piesche, Thorsten J. Maier, Khaled Machaca

**Affiliations:** ^1^Department of Biology and Chemistry, Faculty of Basic Sciences, Universidad Católica del Maule, Talca, Chile; ^2^Biomedical Research Laboratories, Medicine Faculty, Universidad Católica del Maule, Talca, Chile; ^3^Department of Anesthesiology, Intensive Care Medicine and Pain Therapy, Goethe-University Hospital, Frankfurt, Germany; ^4^Department of Physiology and Biophysics, Weill Cornell-Medicine-Qatar, Education City, Qatar Foundation, Doha, Qatar

**Keywords:** ion currents, fertilization, patch-clamp, membrane potential, oocyte maturation, Ca^2+^ signaling

## Abstract

The proper maturation of both male and female gametes is essential for supporting fertilization and the early embryonic divisions. In the ovary, immature fully-grown oocytes that are arrested in prophase I of meiosis I are not able to support fertilization. Acquiring fertilization competence requires resumption of meiosis which encompasses the remodeling of multiple signaling pathways and the reorganization of cellular organelles. Collectively, this differentiation endows the egg with the ability to activate at fertilization and to promote the egg-to-embryo transition. Oocyte maturation is associated with changes in the electrical properties of the plasma membrane and alterations in the function and distribution of ion channels. Therefore, variations on the pattern of expression, distribution, and function of ion channels and transporters during oocyte maturation are fundamental to reproductive success. Ion channels and transporters are important in regulating membrane potential, but also in the case of calcium (Ca^2+^), they play a critical role in modulating intracellular signaling pathways. In the context of fertilization, Ca^2+^ has been shown to be the universal activator of development at fertilization, playing a central role in early events associated with egg activation and the egg-to-embryo transition. These early events include the block of polyspermy, the completion of meiosis and the transition to the embryonic mitotic divisions. In this review, we discuss the role of ion channels during oocyte maturation, fertilization and early embryonic development. We will describe how ion channel studies in *Xenopus* oocytes, an extensively studied model of oocyte maturation, translate into a greater understanding of the role of ion channels in mammalian oocyte physiology.

## Introduction

Historically, the studies of gamete maturation and fertilization have been intimately associated with the regulation of ionic currents in these cells. Some of the earliest discoveries in ion channel biology have been driven by the desire to understand the mechanisms governing fertilization. The calcium (Ca^2+^)-release theory of egg activation, which was conceived based on early experiments in the late 1920s and into the 1930s (Tyler, 1941) has withstood the test of time. Indeed, both single and repetitive Ca^2+^ transient(s), propagating as a Ca^2+^ release wave across the egg is recognized as the trigger for egg activation at fertilization in all species tested to date (Stricker, 1999; Machaca, 2007; Kashir et al., 2013). Further improvements in our understanding of the electrical properties of both oocyte and the egg, have shed light on important processes involved in fertilization including the block to polyspermy. Initial electrophysiology studies determined the essential role of plasma membrane (PM) depolarization to the establishment of a fast block to polyspermy in sea urchin and *Xenopus* oocytes, among other species (Jaffe, 1976; Cross and Elinson, 1980; Jaffe and Cross, 1984).

Oocyte maturation in vertebrates is initiated following the release of the extended meiotic arrest that vertebrate oocytes experience during their growth and development. Oocytes arrest at the prophase stage of meiosis I with the nuclear envelope still intact. During this stage, oocytes grow and accumulate macromolecular components required for fertilization and early embryonic development. Upon hormonal stimulation, oocytes exit this extended meiotic arrest and undergo a complex differentiation pathway that encompasses both a reductionist nuclear division (meiosis) and a comprehensive cytoplasmic reorganization. This prepares the oocyte for the egg-to-embryo transition following fertilization (Smith, 1989; Miyazaki, 1995; Hassold and Hunt, 2001). An important aspect of oocyte maturation is the remodeling of the Ca^2+^ signaling machinery to allow the egg to activate properly at fertilization (Machaca, 2007; Nader et al., 2013). The induction of oocyte maturation ultimately culminates through multiple steps in the activation of maturation promoting factor (MPF). MPF is composed of cyclin dependent kinase 1 (Cdk1), in complex with cyclin B (B-Cdk1), and the associated nuclear kinase Greatwall, also known as microtubule associate threonine like kinase (Gwl/MASTL). MPF is the master regulator of both meitotic and mitotic M-phase (Kishimoto, 2015). Oocyte maturation is complete when oocyte reaches a second arrest in metaphase of meiosis II at which stage they become fertilization-competent and are typically referred to as “eggs” (Smith, 1989; Bement and Capco, 1990). The arrest at metaphase II requires cytostatic factor (CSF) which inhibits the anaphase promoting complex (APC) and prevents progression to Anaphase II (Tunquist and Maller, 2003). The APC is an ubiquitin ligase that tags cyclin B and other regulatory proteins which results in the loss of Cdk1 activity triggering exit from metaphase arrest and allowing progression to anaphase (Schmidt et al., 2005; Inoue et al., 2007; Nishiyama et al., 2007). The activity of ion channels and transporters and their remodeling during oocyte maturation is ultimately governed by this complex signaling cascade. Therefore, oocyte maturation is a cellular differentiation program that prepares the egg for fertilization and for the egg-to-embryo transition processes where ionic conductances play essential roles.

In mammals, fertilization results in the release of a sperm specific phospholipase [phospholipase ζ (PLCζ)] into the egg cytoplasm upon sperm-egg fusion. It has been proposed that PLCζ hydrolyzes not only PM phosphoinositol 4,5 bisphosphate (PIP_2_) but mainly intracellular PIP_2_ (Yu et al., 2012; Swann and Lai, 2016), generating inositol triphosphate (InsP_3_) and diacylglycerol (DAG). InsP_3_ binds to the IP_3_ receptor (IP_3_R) on the endoplasmic reticulum (ER) and triggers the release of Ca^2+^ which mediates egg activation (Saunders et al., 2002). The ultimate role of PLCζ as the trigger for the Ca^2+^ oscillations in mammals was recently elucidated through the generation of a mice lacking *Plcz1* (*Plcz1*^−^). Wild type eggs that were injected with *Plcz1*^−^ sperm were unable to mount Ca^2+^ oscillations. Similar results were observed in experiments of *in-vitro* fertilization (IVF) using *Plcz1*^−^ sperm. Surprisingly, *Plcz1*^−^ mice are fertile, yet they do exhibit subfertility phenotype, suggesting a redundant system for egg activation to assure reproduction in animals (Hachem et al., 2017). The fertilization Ca^2+^ signal takes the form of multiple oscillations that encode egg activation events, including pro-nucleus formation and the transition to embryonic development (Ducibella et al., 2002). In frogs, sea urchin and starfish, fertilization is also associated with a change of resting membrane potential, referred to as the “fertilization potential” which was compared to the action potential in neurons early on. The egg membrane potential undergoes a transient positive shift at fertilization that acts as an electrical polyspermy blockade. The effect of membrane potential depolarization on the polyspermy block was confirmed by holding the membrane potential of an unfertilized egg at positive potentials which prevented fertilization (Jaffe and Cross, 1984). In 2014, a detailed analysis of published data challenged the evidence of the fast block of polyspermy in sea urchin, proposing that a particular organization of the cytoskeleton could determine monospermic fertilization (Dale, 2014). However, the time scale of the electrical fast block would argue against it.

In contrast, hamster eggs showed transients hyperpolarization in response to fertilization (Miyazaki and Igusa, 1981), while mice eggs only showed very small hyperpolarization (3–4 mV), questioning the existence of electrical block to polyspermy in this species (Igusa et al., 1983). It has rather been proposed that the block to polyspermy in mammals is based on the hardening of the *zona pellucida* (ZP). This process is mediated by the protein ovastacin whose function is to cleave *zona pellucida* 2 (ZP2), rendering the ZP resistance to protease digestion and inhibiting sperm binding (Burkart et al., 2012). Notwithstanding, transgenic mice containing a non-cleavable ZP2 and female mice null for ovastacin are both fertile, suggesting an additional mechanism or a combination of different strategies to avoid polyspermy (Bianchi and Wright, 2016).

In this review, we discuss the role of ionic currents at the PM primarily during oocyte maturation and fertilization. These conductances encompass transporters in addition to Cl^−^ channels, K^+^ channels, Ca^2+^ channels, and other channels that are important in mediating fertilization and egg activation. Therefore, it is important to understand the regulation of these conductances as their remodeling contributes to define the competence of the egg to fertilization and undergo the egg-to-embryo transition. Interestingly, the activity of ion channels during the processes of oocyte maturation and fertilization is not limited to channels localizing to the PM but also includes intracellular channels. As a case in point, the role of changes in the IP_3_ Receptor (IP_3_R) activity and localization observed during *Xenopus* oocyte maturation in preparing the egg for fertilization will be discussed.

The role that ionic currents play in the regulation of oocyte maturation is increasingly being recognized. Hence, it is important to elucidate the molecular mechanisms governing their remodeling during maturation and activity at fertilization. Furthermore, comparative studies from different species, as would be discussed herein between *Xenopus* and mammals, provide important insights into the mechanisms governing the regulation of ion channels in gametes.

## Initial measurements of ion channel activity in reproduction

The electrical properties of eggs particularly at fertilization have been of interest to embryologists since membrane potential changes were first recorded in neurons and muscle cells (Hille, 2001). It is now well established that the membrane potential is maintained by differences in ion concentrations between the intra and extra cellular media. The resulting generation of an electrical potential difference across the PM can be measured through electrophysiology. Intracellular voltage and current polarity are defined in relation to the “ground” (zero voltage) reference electrode in the extracellular medium (Ypey and DeFelice, 2000). Original assessments of the egg's membrane potential using standard electrophysiological approaches were performed in eggs from marine species. Although initial attempts using capillary microelectrodes failed, the implementation of intracellular microelectrodes allowed the measurement of resting potential and ion fluxes through the membrane of star fish eggs (Tyler et al., 1956). Invariably, the membrane potential in the resting state or “resting potential” (RP), in all living cells has a negative value expressed as a difference using the bath as the “ground” reference (for example, a neuron RP ranges from −70 to −90 mV). Ion channels transport ions down their concentration gradient, from areas of high to low abundance, generating ion currents that determine, and regulate membrane potential (Hille, 2001; Tosti et al., 2013).

Using an intracellular microelectrode and a conventional extracellular ringer, the RP of mouse eggs was shown to vary depending on the mouse strain (~ −14 to −20 mV). The membrane showed permeability to K^+^ and Na^+^, however, one needs to consider the possibility that these conductances may be affected by damage to the eggs by the electrode impalement procedure (Powers and Tupper, 1974; Hagiwara and Jaffe, 1979). The development of the patch-clamp technique changed the landscape relating to accuracy in data acquisition, allowing the mouse egg's RP and ion channel activity to be measured with minimal damage to the cell (Hamill et al., 1981). Conventional voltage-clamp and patch-clamp measurements indicate that in mouse eggs, the RP ranges between ~ −30 and −50 mV depending on the composition of the extracellular media (Igusa et al., 1983; Peres, 1986; Bernhardt et al., 2015). Using the same methodology, Maeno described a fundamental difference between the action potential of a nerve or muscle cell and the ones measured from oocytes and eggs from the toad. During egg activation, changes in membrane potential were caused by an increased permeability to Cl^−^ ions, whereas action potentials of neurons and muscle cells were driven by changes in Na^+^ and K^+^ conductances (Maeno, 1959). Electrophysiology is the most direct approach to study ion channels and allows direct characterization of these proteins in oocytes and eggs. Below, we describe the properties of ion channels that have been implicated in oocyte maturation and fertilization.

## *Xenopus* vs. mammalian oocytes: comparison of ion channel expression and function

The fluxes of ions during oocyte maturation and at fertilization are mediated by ion channels, transporters and pumps. Most of them localize at the PM; however, intra and intercellular channels are also fundamental players supporting cellular processes. Ca^2+^ is the main signal underlying oocyte maturation and egg activation. Transport of Ca^2+^ through ion channels have been recognized as critical step, for example, to assure egg activation. In this section ion channels expressed in *Xenopus* and mammalian oocytes and eggs will be reviewed. Due to the fundamental role of Ca^2+^ in reproduction, we will focus on Ca^2+^ channels and Ca^2+^ modulated channels.

### Ca^2+^ signaling

Ca^2+^ is a universal second messenger that is fundamental to cellular signaling and homeostasis (Berridge, 2005; Clapham, 2007). The ionic nature of Ca^2+^ makes it unique among second messengers. Agonist activation of particular receptor types triggers the transport of Ca^2+^ into the signaling compartment (the cytoplasm) through channels, transporters and/or pumps. Cells support Ca^2+^ signaling by maintaining low cytoplasmic Ca^2+^ levels at rest (~100 nM). During the rising phase of a Ca^2+^ signal, Ca^2+^ flows into the cytoplasm either from the endoplasmic reticulum (ER) which concentrates Ca^2+^ at 250–600 μM, or from the extracellular space, where Ca^2+^ concentrations are typically 1–2 mM (Clapham, 1995; Demaurex and Frieden, 2003). ER localized non-selective cation channels, typically IP_3_ Receptors (IP_3_R) or Ryanodine Receptors (RyR), mediate Ca^2+^ release from the ER. Both IP_3_Rs, and RyRs have been detected in rodent oocytes (Miyazaki et al., 1992; Kline and Kline, 1994). *Xenopus* oocytes, in contrast, express only a single isoform of Ca^2+^ release channels, the type 1 IP_3_R (Parys et al., 1992; Parys and Bezprozvanny, 1995). Both mammals and *Xenopus* eggs require IP_3_-dependent Ca^2+^ release from the ER for egg activation at fertilization (Larabell and Nuccitelli, 1992; Miyazaki et al., 1992, 1993; Swann, 1992; Nuccitelli et al., 1993; Kline and Kline, 1994; Jones et al., 1995; Runft et al., 2002).

Ca^2+^ is the universal signal for egg activation at fertilization in all sexually reproducing species tested (Stricker, 1999; Whitaker, 2006). Thus, different species have evolved elaborate strategies to safeguard reproductive isolation. These include preventing the fusion of gametes from divergent species even if they are evolutionarily similar (Vieira and Miller, 2006). The versatility and diversity of Ca^2+^ in mediating a plethora of physiological responses make it an ideal second messenger to induce egg activation at fertilization. Ca^2+^ is able to signal across broad spatial (from the nm to cm scales) and temporal (μsec to h) ranges (Berridge et al., 2000). Ca^2+^ mediates cellular responses ranging from the rapid and localized like neurotransmitter release, to the slow and spatially spread out, such as the activation of development at fertilization.

Immature fully-grown vertebrate oocytes in the ovary are unable to support the egg-to-embryo transition. Eggs acquire the ability to be activated at fertilization during oocyte maturation. A major component of acquiring this competence is the remodeling of the Ca^2+^ signaling machinery, primarily the modulation of Ca^2+^ channels and transporters function during oocyte maturation. This has been best studied in *Xenopus* (Machaca, 2007) and mammalian oocytes, and will be reviewed briefly below.

### Ca^2+^ release

The frog egg is an exemplary model for the study of Ca^2+^ dependent egg activation processes including block to polyspermy and the release of metaphase II arrest to complete meiosis (Stricker, 1999; Runft et al., 2002; Whitaker, 2006). Immediately after sperm fusion, the Ca^2+^ transient activates Ca^2+^-activated Cl^−^ channels (CaCC), resulting in membrane depolarization and the so called “fast electrical block to polyspermy” (Machaca et al., 2001). This is followed by cortical granule fusion which is also a Ca^2+^-dependent event. The cortical granule reaction results in modification of the egg extracellular matrix and a more permanent block to polyspermy (Grey et al., 1974; Wolf, 1974). Polyspermy block in mammals is hypothesized to occur through a similar Ca^2+^ evoked cortical granule release process (Abbott and Ducibella, 2001), in addition to other mechanisms.

Following the establishment of the polyspermy block, Ca^2+^ signaling then induces the egg to exit from metaphase II. The exit occurs by activating Ca^2+^-calmodulin-dependent protein kinase II (CaMKII) to start the egg activation processes (Lorca et al., 1993). CaMKII phosphorylates Emi2, an essential component of CSF (Schmidt et al., 2006). Emi2 is a direct inhibitor of APC. CaMKII-mediated degradation of Emi2 releases the oocyte from CSF-mediated metaphase II arrest, allowing anaphase II to proceed (Liu and Maller, 2005; Rauh et al., 2005; Tung et al., 2005). This activates the APC, leading to ubiquitination and degradation of Cyclin B. Degradation of Cyclin B, in turn, downregulates MPF activity and allows meiosis to reach completion (Morin et al., 1994). The fertilization-induced Ca^2+^ signal also activates the Ca^2+^-dependent phosphatase, calcineurin, which reinforces APC activation and the degradation of Cyclin B (Mochida and Hunt, 2007; Nishiyama et al., 2007). The Ca^2+^ transient induced by fertilization encodes sequential cellular rearrangements that are critical for egg activation.

### IP_3_ receptor

IP_3_ receptors (IP_3_R) are tetrameric channels with the structure of each subunit consisting of six transmembrane domains, a p-loop and a large cytoplasmic domain representing the bulk of the protein. They are non-selective cation channels that are Ca^2+^-permeant. Given the large Ca^2+^ gradient established across the ER membrane, IP_3_R gating results in Ca^2+^ release from intracellular ER Ca^2+^ stores (Parys and Bezprozvanny, 1995; Foskett et al., 2007). The IP_3_R has three isoforms, type 1, 2, and 3. All three isoforms are expressed in mammalian oocytes and eggs, with the type I isoform being the most dominant (Wakai et al., 2011). The IP_3_R is modulated by Ca^2+^ and IP_3_ and requires the binding of both for the conduction path to open (Taylor and Tovey, 2010). In most vertebrates, the IP_3_R is responsible for the fertilization-induced Ca^2+^ transient. In mammals, an initial first large Ca^2+^ transient is observed upon fertilization, similar to event observed in frog eggs. Unique to mammals however, are the multiple Ca^2+^ oscillations that follow this large transient, and last for several hours (Kline and Kline, 1992; Mohri et al., 2001). The phosphorylation of the IP_3_R was proposed to modulate Ca^2+^ fluxes from the ER. Initial theories proposed that phosphorylation impacted the activity of the IP_3_R during oocyte maturation by promoting Ca^2+^ transport at fertilization (Fujiwara et al., 1993; Mehlmann and Kline, 1994; Terasaki et al., 2001; Machaca, 2004; Zhang et al., 2015); (Table 1). In mouse IP_3_R, several serine/threonine residues have been identified as targets for phosphorylation during oocyte maturation (Westendorf et al., 1994). Cell cycle kinases such as mitogen-activated protein kinase (MAPK), extracellular signal-regulated kinase (ERK) and Cdk1 have been shown to phosphorylate the receptor (Bai et al., 2006; Lee et al., 2006; Zhang et al., 2015). Kinase motifs include the Cdk1 consensus sites [S^421^, T^799^, S^2147^] (Nigg, 1991; Malathi et al., 2003; Wakai et al., 2012) and the ERK site [S^436^] (Bai et al., 2006; Lee et al., 2006). In addition, several other kinases have been reported to phosphorylate the IP_3_R including protein kinase A (PKA, [S^1589^, S^1755^, and T^930^] Ferris et al., 1991; Haun et al., 2012; Xu and Yang, 2017), protein kinase G (PKG, same sites as PKA), protein kinase C (PKC, Vanderheyden et al., 2009) and protein kinase B (PKB or Akt, [S^2681^] Vanderheyden et al., 2009. Other kinases such as CaMKII and Rho kinases also mediate phosphorylation of the IP_3_R (Vanderheyden et al., 2009). Originally, it was suggested that phosphorylation modulates the function of the IP_3_R by sensitizing its release of Ca^2+^ at fertilization (Fujiwara et al., 1993; Mehlmann and Kline, 1994; Terasaki et al., 2001; Machaca, 2004; Zhang et al., 2015) (Table 1). In *Xenopus*, this sensitization was thought to be due to an increased affinity of the IP_3_R for IP_3_ (Machaca, 2004; Ullah et al., 2007). However, phosphorylation of the IP_3_R does not ultimately result in an increased affinity for IP_3_ (Sun et al., 2009). High constitutive PKA activity in the oocyte is required to maintain meiotic arrest, resulting in basal phosphorylation of IP_3_R PKA sites. This phosphorylation is unaltered during maturation (Sun et al., 2009). The IP_3_R is also phosphorylated at additional sites that match the MAPK and/or the Cdk1. Activation of MAPK or Cdk1 is required for sensitizing IP_3_-dependent Ca^2+^ release during oocyte maturation (Sun et al., 2009). The MAPK/Cdk1 consensus phosphorylation sites altered during oocyte maturation are T931, T1136, and T1145 (Sun et al., 2009). Surprisingly though, phosphorylation of the IP_3_R on at least one of those residues decreased its affinity to IP_3_ rather than the expected increase in affinity (Haun et al., 2012). This observation argues against phosphorylation being the primary driver of the increased sensitivity of the IP_3_R for IP_3_ observed during maturation. As it turns out, the mechanism is much more complex and elegant. The ER suffers dramatic remodeling that is dependent on the kinase cascades activated during maturation, in the process of “geometric sensitization” (Sun et al., 2011). The ER remodeling results in the formation of large patches of convoluted membranes highly enriched with IP_3_Rs orienting in close apposition to each other (Sun et al., 2011). IP_3_Rs within these ER patches exhibit a significantly enhanced sensitivity to IP_3_ compared to IP_3_Rs within the adjacent ER, despite the fact that IP_3_Rs exchange freely between the patches and adjacent ER (Sun et al., 2011). Thus, the sensitization of IP_3_Rs during oocyte maturation is due to ER remodeling, and the enhanced Ca^2+^-induced Ca^2+^ release evoked by the close apposition of IP_3_Rs (geometric sensitization) (Sun et al., 2011).

**Table 1:** Channel expression and function in *Xenopus* and mouse oocytes and eggs.

**Channel**	***Xenopus***	**Mouse**
**INTRACELLULAR**		
IP_3_R1	Oocytes and eggs showed responses to IP_3_. IP_3_R in mature eggs is more sensitive to IP_3_ than it is in oocytes (Terasaki et al., 2001; Machaca, 2004; Sun et al., 2009, 2011)	Expressed at GV oocytes and MII eggs. Increases activity during oocyte maturation (Mehlmann and Kline, 1994)
**PLASMA MEMBRANE (PM)**		
CRAC (ORAI+STIM)	Inactivates during oocyte maturation (Machaca and Haun, 2000, 2002; Yu et al., 2009, 2010)	Inactivates during oocyte maturation (Cheon et al., 2013; Lee et al., 2013). *Orai* and *Stim* KO animals are fertile (Bernhardt et al., 2017)
T type Ca^2+^ channel	Not reported	Expressed in GV and MII. KO animals are fertile (Chen et al., 2003; Bernhardt et al., 2015)
TRPV3	Not reported	Expressed at MI oocytes and MII eggs. KO animals are fertile (Cheng et al., 2010; Carvacho et al., 2013)
TRPM7	Not reported	TRPM7-like currents are expressed at GV, MII and in 2-cell embryos (Carvacho et al., 2016)
Ca^2+^ activated chloride channels (CaCC)	Expressed in eggs. Responsible for fast electrical block to polyspermy (Cross and Elinson, 1980), regulates resting membrane potential (Kuruma and Hartzell, 2000), and length of microvilli (Courjaret et al., 2016)	Expressed in embryos (Li et al., 2007)
Swell-activated Cl^−^ channels	Not reported	Functionally expressed in MII eggs and embryos (Kolajova et al., 2001)
Voltage activated K^+^ channels	Expressed in eggs (Tokimasa and North, 1996)	Reported in MII eggs (Day et al., 1993)
Connexins (Cx37 and Cx43)	Not reported	Cx37 KO animals are infertile (Simon et al., 1997) Ovaries lacking Cx43 contain oocytes that cannot be fertilized (Ackert et al., 2001)

Another Ca^2+^ transport protein, the PM Ca^2+^-ATPase (PMCA), is also modulated during oocyte maturation. In *Xenopus* oocytes, PMCA localizes to the PM where it contributes to Ca^2+^ extrusion. This activity supports the return of cytoplasmic Ca^2+^ concentration to baseline levels following a Ca^2+^ transient at fertilization (El Jouni et al., 2005, 2008). During oocyte maturation, the PMCA is removed from the PM through an internalization process that places them in an intracellular vesicular pool (El Jouni et al., 2005, 2008). PMCA internalization during meiosis is dependent on its N-terminal cytoplasmic domain and on MPF activation (El Jouni et al., 2008). Furthermore, several lines of evidence argue that PMCA internalization goes through a lipid-raft endocytic pathway (El Jouni et al., 2008).

### Calcium channels

#### Store-operated Ca^2+^ entry, SOCE

Ca^2+^ influx into cells is mediated by a diverse population of Ca^2+^ transport proteins exhibiting significant diversity in their gating and activation mechanisms. Ca^2+^ channels at the PM can be gated by voltage, ligand, second messengers, store depletion, or physically through protein-protein interactions. In vertebrate oocytes the predominant Ca^2+^ influx pathway appears to be through store-operated Ca^2+^ entry (SOCE). SOCE is directly regulated by the level of Ca^2+^ in intracellular ER stores (Kline and Kline, 1992; Hartzell, 1996; Machaca and Haun, 2000, 2002). Emptying of intracellular ER stores can be triggered using thapsigargin, an irreversible inhibitor of the sarcoplasmic reticulum/ER Ca-ATPase (SERCA). The pathway through which Ca^2+^ exits the ER into the cytoplasm following thapsigargin treatment has not yet been identified. Thapsigargin-induced depletion of the ER Ca^2+^ store activates the influx of extracellular Ca^2+^ into the cytoplasm of unfertilized eggs (Kline and Kline, 1992). When ER Ca^2+^ stores have been significantly depleted by the persistent presence of thapsigargin, sperm are no longer capable of triggering Ca^2+^ oscillations (Kline and Kline, 1992). Evidence that Ca^2+^ influx is essential for the maintenance of oscillations triggered by sperm was demonstrated by addition of BAPTA to the extracellular media. BAPTA is a membrane impermeable Ca^2+^ chelator which prevented the generation of oscillations by the sperm (Kline and Kline, 1992). Additionally, the frequency of Ca^2+^ oscillations can be modulated by changing the external concentration of Ca^2+^ (Shiina et al., 1993). Thus, extracellular Ca^2+^ is an important source to support Ca^2+^ oscillations.

SOCE or Ca^2+^ release-activated Ca^2+^ channels (CRAC), were first described in immune cells where they have been shown to be critical for their activation. Accordingly, defects in SOCE in humans are associated with severe immunodeficiencies (Bogeski et al., 2010). SOCE is mediated through the interactions of ER Ca^2+^ sensors, stromal interaction molecule (STIM), with ORAI ion channels at the PM. STIM proteins cluster in the ER following store depletion, localizing to ER-PM junctions where they physically recruit and interact with ORAI proteins to gate their pore open (Lewis, 1999). ORAIs are 4 pass transmembrane proteins that form highly Ca^2+^-selective channels (Prakriya et al., 2006; Vig et al., 2006). STIM has two homologs: STIM 1 and 2; and ORAI has three family members, ORAI1, 2 and 3 (Shim et al., 2015).

During *Xenopus* oocyte maturation, SOCE is completely inactivated (Machaca and Haun, 2000). This inactivation is essential for the remodeling of Ca^2+^ signaling pathways to enable the generation of the specialized fertilization-specific Ca^2+^ transient that encodes the egg-to-embryo transition (Machaca, 2007; Nader et al., 2013) (Table 1). As discussed above, this Ca^2+^ signal at fertilization is the spark that induces egg activation (Fontanilla and Nuccitelli, 1998). *Xenopus* oocyte SOCE downregulation may represent a safety mechanism to prevent premature activation due to spontaneous Ca^2+^ influx. *Xenopus* eggs are ovulated in pond water of uncontrolled ionic content. Indeed, all ionic currents across the egg PM tend to be downregulated, with the exception of the Ca^2+^-activated Cl channels which are required for the block to polyspermy as discussed below (Nader et al., 2013).

The mechanisms governing SOCE inhibition during *Xenopus* oocyte maturation have been studied in detail. The activation of MPF is required for SOCE inhibition during maturation (Machaca and Haun, 2002). This results in the internalization of ORAI1 into a Rab5-positive endosomal compartment through a caveolin and dynamin-dependent endocytic pathway (Yu et al., 2009, 2010). STIM1 does maintain its ability to interact with ORAI1 in *Xenopus* eggs, however, in mature eggs; ER store depletion does not lead to STIM1 clustering. Clustering is a pre-requisite for STIM stabilization in the cortical ER (Yu et al., 2009). Although stim1 is phosphorylated during oocyte maturation, this phosphorylation does not modulate STIM1 function or its inhibition during meiosis. Mutant STIM proteins that cannot be phosphorylated are unable to rescue SOCE downregulation in *Xenopus* eggs, even when co-expressed with an *Orai1* mutant that cannot be internalized (Yu et al., 2009; 2010).

**Figure 1:**
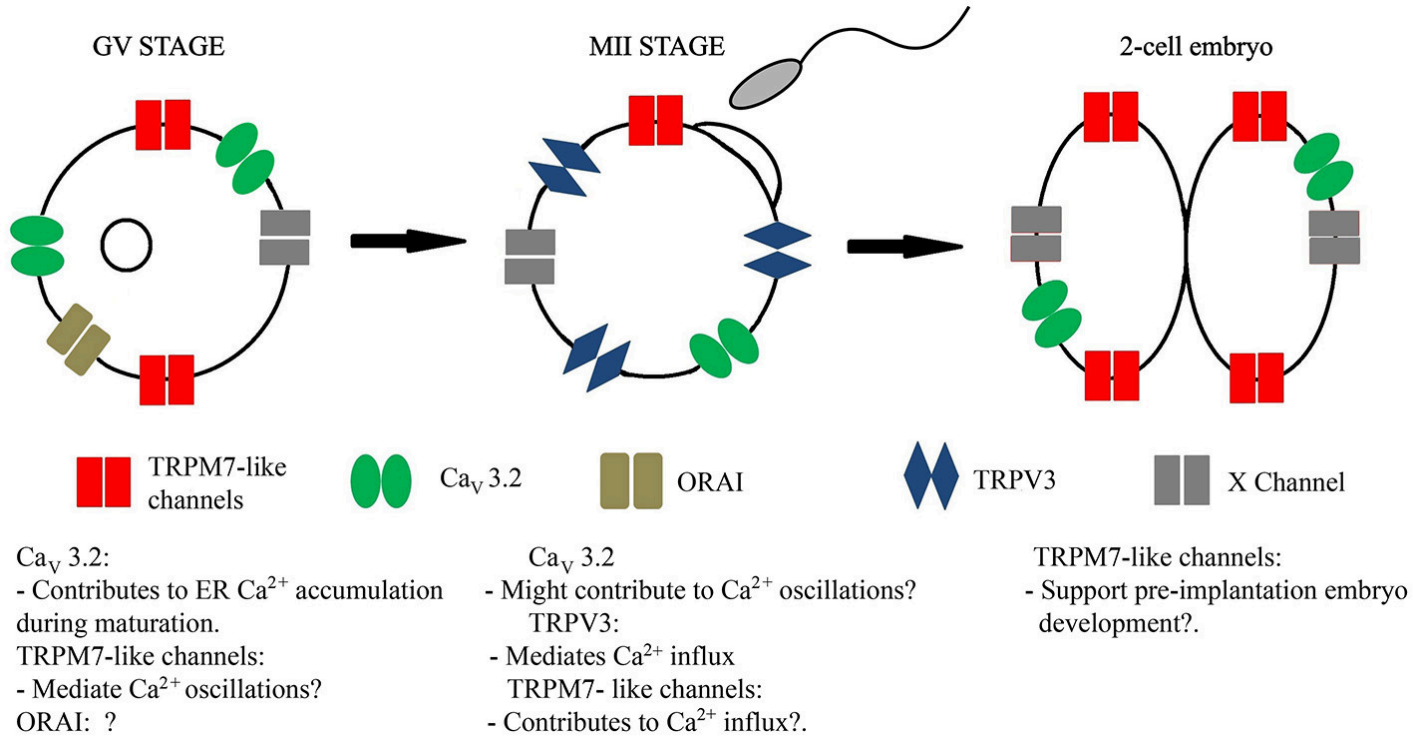
Schematic representation of the Ca^2+^ channels reported to be expressed in GV stage, MII, and 2-cell embryo. GV, germinal vesicle.

Expression of SOCE components in mammals has been shown at the mRNA levels as well as by immunocytochemistry and western blotting in mouse oocytes (Gomez-Fernandez et al., 2009; Cheon et al., 2013) and porcine eggs (Koh et al., 2009; Wang et al., 2012). However, in some cases, the specificity of the antibodies used requires additional confirmation. Further, the function of SOCE during mouse fertilization remains controversial and may play a minor role. In a MII egg study, STIM1 was found to form discrete patches co-localizing with an ER marker prior to fertilization. This organization changed following Ca^2+^ depletion showing high co-localization with ORAI1. Thus, a role for SOCE in Ca^2+^ signaling during fertilization was suggested (Gomez-Fernandez et al., 2009, 2012). However, the size of the large STIM patches observed in these studies was not consistent with the patch size noted in other cells. In addition, specific inhibitors of SOCE did not disrupt Ca^2+^ oscillations induced by fertilization (Miao et al., 2012; Carvacho et al., 2013). Finally, experiments tracking the expression of exogenously-tagged STIM1 and ORAI1 proteins found that SOCE downregulation during oocyte maturation was mainly due to reorganization of STIM and an internalization of ORAI1 (Cheon et al., 2013; Lee et al., 2013) (Table 1 and Figure 1).

One of the main issues regarding the functional expression of CRAC channels in mouse eggs is the lack of electrophysiological evidence. CRAC channels have a small unitary conductance such that at physiological concentration of Ca^2+^ (2 mM) the single channel conductance of CRAC channels is ≤ 9 fS, and at 20 mM Ca^2+^ it is 18–24 fS (McNally and Prakriya, 2012). Given the magnitude of the expected whole-cell current and the background currents in eggs, detection of CRAC channels activity would be technically challenging. Additionally, protocols to empty intracellular Ca^2+^ stores weaken the stability of the patch-clamp seal during electrophysiological measurements. Final confirmation that the function of STIM1 and ORAI1 are not required for fertilization and the egg-to-embryo transition came from the generation of oocyte-specific knockout mouse line for *Stim1/2* and the study of the global KO for *Orai1* (Vig et al., 2008). These lines showed no fertility defects and the eggs showed a normal pattern of Ca^2+^ oscillations at fertilization (Bernhardt et al., 2017).

There are reports that SOCE in some cell types can be mediated by interaction between STIM1 and members of the Transient Receptor Potential (TRP) channel family, particularly from the TRP canonical (TRPC) subfamily (Zeng et al., 2008; Yuan et al., 2009). TRPCs channels have been proposed to interact with STIM1 and act as store-operated channels. TRPC can form complexes with ORAI, however, it has been shown that ORAI channels are functional in absence of TRPCs (Birnbaumer, 2015). Additionally, the heptaKO females for TRPCs (C1–C7) channels are fertile (Birnbaumer, 2015) and eggs from these animals showed normal Ca^2+^ oscillations (Bernhardt et al., 2017). These evidences rule out a role for TRPCs in Ca^2+^ influx. The fact that a *Stim 1/2* KO mouse is fertile argues that SOCE is not essential for fertilization in this species or that there are redundant ionic pathways in the eggs able to refill the stores sufficiently even in the absence of SOCE. The latter scenario seems more attractive especially that SOCE is detectable by Ca^2+^ imaging in mouse eggs despite the fact that it is downregulated during oocyte maturation (Cheon et al., 2013; Lee et al., 2013). Despite the strong evidence against a role for STIM and ORAI at fertilization, their overexpression in mouse oocyte has been shown to disrupt early embryonic development (Lee et al., 2013). Thus, the regulation of Ca^2+^ influx during fertilization is critical for normal egg-to-embryo transition.

In pig eggs, the evidence that STIM1 plays a role in refilling intracellular Ca^2+^ stores and fertilization is more direct: knockdown of *Stim1*, using RNAi, abolished thapsigargin-induced Ca^2+^ influx (Koh et al., 2009), sperm-induced Ca^2+^ oscillations and affected embryo development (Lee et al., 2012). Manipulating the expression of *Orai1* also had consequences on store-operated Ca^2+^ influx. Overexpression of *Orai1* disrupted the oscillations triggered by fertilization. Downregulation of *Orai*1 abolished Ca^2+^ oscillations and diminished the rate of blastocyst formation (Wang et al., 2012; Machaty et al., 2017). Using FRET (Fluorescence Resonance Energy Transfer) it was shown that mVenus-*Stim1* and mTurquoise2-*Orai*, constructs that were injected in eggs, interacted following a cyclic pattern in response to store depletion during sperm-induced Ca^2+^ oscillations (Zhang et al., 2018). This interaction suggests a role for SOCE during fertilization in pigs. Overexpression of proteins that accumulate at the PM or in its vicinity can cause non-specific effects on the function of channels. Therefore, electrophysiological detection of native CRAC current in these eggs will be necessary to confirm its functional expression and clarify its role in the early stage of development.

#### Voltage gated Ca^2+^ channels, Ca_V_ channels

Voltage-gated Ca^2+^ (Ca_V_) channels are transmembrane proteins that are organized in four domains (I–IV) with each domain having six transmembrane segments (S1–S6). Ca_V_ channels can be classified into two groups according to the voltage changes required for activation: high-voltage activated (HVA) channels and low-voltage activated (LVA). Ca_v_ 1.1–1.4 (L-type current) and Ca_v_2.1–2.3 (P/Q, N, and R type) belong to the HVA group, while Ca_v_3.1–3.3 (T-type current) to the LVA group. Between other functions, voltage-gated Ca^2+^ channels are responsible for initiation of synaptic transmission, hormone secretion, and excitation-contraction coupling (Hille, 2001; Catterall, 2011; Ramirez et al., 2017).

In 1977, using the voltage-clamp technique, an inward Ca^2+^current was described in mouse eggs. The recordings were done in 20 mM external Ca^2+^ and the inward current increased in response to depolarizing steps. The peak current was measured at ~ −15 mV and it showed a dramatic reduction at temperatures below 20° C. Replacement of external Ca^2+^ by Sr^2+^ or Ba^2+^ retained the biophysical characteristics of the channel. Sea urchin and tunicate eggs also showed an inward Ca^2+^current but with different inactivation time and permeability ratio than the one recorded in mouse eggs (Okamoto et al., 1977). In mouse eggs, using an improved set-up, the maximal current was at ~ −30 mV and its reversal potential was ~+50 mV (Peres, 1987). Similar characteristics were found using the patch-clamp technique (Kang et al., 2007; Bernhardt et al., 2015). Additional recordings of mouse ovarian oocytes using single-glass microelectrodes showed inward currents carried by monovalent cations. These currents were blocked by Ca^2+^ channel antagonists and were insensitive to tetrodotoxin (TTX), confirming the functional presence of Ca^2+^ channels before ovulation (Yoshida, 1983). After fertilization, the voltage-gated Ca^2+^ channel varies in magnitude but not in kinetics or selectivity (Yamashita, 1982; Day et al., 1998; Kang et al., 2007). Voltage-gated Ca^2+^ channels were also described in hamster (Georgiou et al., 1984) and bovine eggs (Tosti et al., 2000). Comparable voltage-gated channels were recorded in ascidians and mollusks (Gallo et al., 2013; Tosti et al., 2013). Later, biophysical characterizations of the Ca_V_ current in mouse eggs assigned it molecularly as a T-type 3.2, voltage-gated Ca^2+^ channel (Kang et al., 2007) (Table 1). Surprisingly, even when the T-type current is prominent in mouse eggs (peak reaches ~1.5 nA in MII eggs, in 2 mM Ca^2+^), mice null for this channel*, Cacna1h*^−/−^ (Chen et al., 2003), showed only marginal subfertility (Bernhardt et al., 2015). These results suggest that the function of this channel may be to support Ca^2+^ influx during the germinal vesicle arrest (GV; Figure 1) and during maturation. Ca_V_ channels would then contribute to fill the intracellular ER Ca^2+^ stores (Figure 1) and maintain Ca^2+^ homeostasis in preparation for fertilization (Bernhardt et al., 2015).

Native Ca_V_ channels of an unspecified type are expressed in *Xenopus* oocyte. The conductance was smaller than most conductances recorded in these cells (Dascal et al., 1986). Their function remains unknown.

#### Transient receptor potential (TRP) channels

The TRP channels are a family of cationic non-selective channels that are known as cellular sensors. TRP channels are modulated by common second messengers such as PIP_2_ and intracellular Ca^2+^ but also respond to more general stimuli such as temperature, pH and osmolarity, among others. TRP channels are tetramers where each subunit includes six transmembrane domains (S1–S6) and a p-loop that defines the channel pore (Wu et al., 2010).

TRP channels expression in mouse eggs have been recently validated, despite the fact that a temperature-dependent, outward current was reported in mouse eggs nearly 40 years ago (Okamoto et al., 1977). Confirmation of expression and function of TRP channels in mature eggs was accomplished using patch-clamp and Ca^2+^ imaging (Carvacho et al., 2013). The first TRP channel reported in eggs was TRPV3 channel. TRPV3 is a member of the vanilloid subfamily of the TRP family which is highly expressed in skin. TRPV3 is activated by stimuli such as temperature and plant-derivatives compounds (e.g., carvacrol and eugenol). Other traditional Ca^2+^ channel blockers such as 2-aminoethoxydiphenyl borate (2-APB) are also modulators of TRPV3 (Peier et al., 2002; Smith et al., 2002; Xu et al., 2002; Hu et al., 2004; Lee et al., 2016). TRPV3, similar to many other members of the TRP family can act downstream of G-protein coupled receptor activation (Yang and Zhu, 2014). In mouse eggs a combination of electrophysiological recording and using the agonist 2-APB showed that TRPV3 current is detectable in WT eggs but not in those from *TprV3*^−/^^−^ animals (Carvacho et al., 2013). The current developed progressively during oocyte maturation, reaching the highest level in eggs at the MII stage. TRPV3 can mediate Ca^2+^ influx (Figure 1) which causes an intracellular Ca^2+^ rise capable of promoting parthenogenesis in ovulated eggs. Parthenogenesis and Ca^2+^ oscillations can be triggered artificially by incubation of the ovulated eggs in a strontium-containing media (Whittingham and Siracusa, 1978). TRPV3 channels are responsible for the influx of strontium (Sr^2+^) into eggs. Incubation of mouse eggs in Sr^2+^ containing media has been used for years to induce artificial activation. Sr^2+^-induced egg activation is a procedure that is widely used for animal cloning (Wakayama et al., 1998). Despite the expression of TRPV3 current in MII eggs, *TrpV3*^−/−^ females are fertile (Cheng et al., 2010; Carvacho et al., 2013). These studies suggest the function of an additional channel mediating Ca^2+^ influx, or as discussed above, the concerted function of multiple redundant pathways to ensure proper egg activation. Consistently, a recent study showed the presence of the *TrpV3* transcript in human oocytes. The TRPV3 agonists 2-APB and carvacrol were shown to promote egg activation suggesting functional expression of channels, despite the fact that Sr^2+^ fails to induce activation in human eggs (Lu et al., 2018). One explanation to these results could be that human TRPV3 channels display a different sequence of ion selectivity than mouse TRPV3. Alternatively, the level of localization of TRPV3 at the PM could be too low to allow sufficient Sr^2+^ to induce intracellular Ca^2+^ release. Direct assessments of functional expression of TRPV3 channels in human oocytes would help to solve differences between mouse and human eggs.

Recently, another member of the TRP channels family, TRPM7, was found to be expressed in mouse eggs. TRPM7 belongs to the subfamily of melastatin and exhibits a ubiquitous tissue distribution. *Trpm7*^−/−^ global knock-out is embryonic lethal. Embryos *Trpm7*^*fl*/*fl*^ (Cre-ER) derived from a tamoxifen-inducible (Cre-ER) transgenic line bred to *Trpm7*^*fl*/*fl*^ died earlier than E14.5 (Jin et al., 2008, 2012). One possible interpretation of these results is that TRPM7 is expressed in eggs and/or embryos. Accordingly, a monovalent cationic outward current with the characteristics of a TRP channel was recorded in mouse TRPV3 KO eggs using whole cell patch clamp. The channel responds to TRPM7 agonists and blockers. TRPM7-like current was also detected in 2-cell stage embryos (Table 1 and Figure 1). The chemical suppression of the channel hours after fertilization reduced progression to the blastocyst stage, in agreement with a possible role of TRPM7 in pre-implantation embryo development (Carvacho et al., 2016). Using the same blocker (NS8593), Williams's group showed that eggs treated with NS8593 and fertilized *in-vitro* display impaired Ca^2+^ oscillations (Bernhardt et al., 2017). Future studies following the generation of an oocyte-specific KO for TRPM7 will provide more specific answers about the contribution of TRPM7 during fertilization and/or pre-implantation embryo development.

Expression of TRP channels have been reported in *Xenopus* oocytes. In contrast to mouse oocytes, *Xenopus* TRP channels seem to be inactive. TRPC1 protein was detected by western blot and immunolocalization, and it was suggested to underlie SOCE in *Xenopus* oocytes (Bobanovic et al., 1999). However, the function of the TRPC1 protein as channel is debatable (Wu et al., 2010). Using RT-PCR and western blot, a *Xenopus* homolog of TRPV5/6, xTRPV6, was found in *Xenopus* oocytes (Courjaret et al., 2013). xTRPV6 channel is not active at PM, although it has been suggested that its functional expression is modulated by interacting with TRPC1 (Schindl et al., 2012; Courjaret et al., 2013).

### Chloride channels

#### Ca^2+^-activated Cl^−^ channels

The *Xenopus* egg is ~1.2 mm in diameter allowing ample membrane area for sperm entry. The slow fusion of cortical granules induced by the fertilization-specific Ca^2+^ signal and the time required for the released enzymes to modify the egg extracellular matrix are too long to prevent additional sperm from entering the egg after the first sperm-egg fusion event. Therefore, *Xenopus* eggs have evolved a fast electrical block to polyspermy that is dependent on the Ca^2+^-dependent gating of Ca^2+^-activated Cl^−^ channels (CaCC) (Table 1). CaCC depolarize the cell membrane thus preventing additional sperm from fusing with the egg (Cross and Elinson, 1980; Jaffe et al., 1983). Interestingly, early studies have shown that simply incubating eggs in media with high Cl^−^ or replacing Cl^−^ with other anions such as I^−^ or Br^−^ lead to polyspermy (Bataillon, 1919; Grey et al., 1982). In retrospect, the effects of these ion substitutions on polyspermy are expected. CaCC induces membrane depolarization by conducting Cl^−^ out of the cell, thus a high extracellular Cl^−^ will inhibit Cl^−^ efflux and membrane depolarization, promoting polyspermy.

CaCC-mediated currents are the predominant currents in *Xenopus* oocytes and are required to maintain the oocyte resting membrane potential (Kuruma and Hartzell, 2000). CaCC are encoded by TMEM16A also known as Anoctamin 1 or Ano1 (Schroeder et al., 2008; Yang et al., 2008). In *Xenopus* oocytes, CaCC interact with ERM proteins to regulate the length of microvilli and the membrane surface area (Courjaret et al., 2016; Table 1). CaCC are activated in response to the sperm-induced Ca^2+^ release at fertilization. CaCC activation and membrane depolarization can be replicated in the egg using different Ca^2+^ sources. Functional assays include pricking the egg and injecting Ca^2+^ or IP_3_ directly on the egg. Another alternative is treating the egg with Ca^2+^ ionophores (Wolf, 1974; Cross, 1981; Busa et al., 1985; Machaca et al., 2001).

CaCCs are also expressed in mouse embryos (Table 1). The trophic factor platelet activating factor, PAF (1-*o*-alkyl-2-acetyl-*sn*-glycerol-3-phosphocoline) has been shown to cause activation of a protein G coupled receptor, phospholipase C and phosphatidylinositol 3-kinase (PIK3). PAF induces transient increases in intracellular Ca^2+^, Ca^2+^ influx, and an anion-driven outward current. The outward current was blocked by niflumic acid (NFA), a selective inhibitor of CaCCs, confirming the identity of the channel. Treatment of 1-cell stage embryos with NFA significantly reduced development to blastocyst stage (Li et al., 2007, 2009), arguing a role for CaCC in early mammalian embryonic development.

#### Swell-activated Cl^−^ channels and transporters

The cell volume regulation is a process that is highly controlled during embryo development. The cell volume regulation is mediated by the activity of swell-activated Cl^−^ channels, which are expressed and active in mouse eggs. Early mouse embryos express chloride channels that are permeable to organic osmolytes and whose expression was shown to be cell-cycle dependent (Kolajova et al., 2001) (Table 1). Mouse zygotes can recover from swelling by activating these channels to release intracellular osmolytes out of the cell. Swell-activated channels are active during meiotic maturation and until the 8-cell or morula stage (Seguin and Baltz, 1997; Kolajova et al., 2001). Nevertheless, later studies showed that regulation of cell volume in pre-implantation embryos is a more complex phenomenon that involves more than one type of channels. These include the Na^+^/H^+^ (NHE1) and the ${\text{HCO}}_{3}^{-}$/Cl^−^ (AE2) exchangers (Baltz and Zhou, 2012). Remarkably, these exchangers are inactive during meiotic maturation and are activated after fertilization. Other proteins involved in cell volume regulation after ovulation include the GLTY1 glycine transporter (Steeves et al., 2003) and the betaine and proline SIT1 transporter. SIT1 transporter regulates the accumulation of the organic osmolyte betaine after fertilization and it is mostly active in the 1- and 2-cell stages, whereas GLTY1 seems to be functionally active until the 4-cell stage (Baltz and Zhou, 2012).

### Potassium channels

#### Ca^2+^ activated potassium channels (K_(Ca)_)

Ca^2+^-activated potassium channels (K_(Ca)_) are tetramers and each subunit has six or seven transmembrane domains. They are divided in three groups depending of their unitary conductance: Big conductance (BK), intermediate conductance (IK) and small conductance (SK). K_(Ca)_ channels are ubiquitously expressed in nearly every vertebrate excitable cell (Hille, 2001).

Fertilization in hamster eggs is marked by hyperpolarization spikes (Miyazaki and Igusa, 1981) that were related with the activity of K_(Ca)_ channels (Miyazaki and Igusa, 1982). The periodic hyperpolarizing pulses during fertilization reach to −70 to −80 mV from a resting potential of −30 mV. They were abolished by injection of the Ca^2+^ chelator EGTA into eggs, suggesting K_(Ca)_ channels function (Georgiou et al., 1983; Igusa et al., 1983). The hyperpolarization responses after fertilization in mouse eggs are Ca^2+^ independent smaller in magnitude than those observed in hamster eggs. Thus, a different pool of channels activity during fertilization between the two species is suggested (Igusa et al., 1983).

Unfertilized human eggs showed hyperpolarization and increased basal current, following an injection of sperm factor, thimerosal (Homa and Swann, 1994), or in response to the Ca^2+^ ionophore A23187. Application of the ionophore in unfertilized eggs activated a bell-shaped current that was blocked by iberiotoxin, a selective blocker of BK channels. In agreement with K_(Ca)_ current, preloading oocytes with EGTA inhibited the current triggered by microinjection of IP_3_. Therefore, the mechanism of hyperpolarization in human eggs seems to be similar to hamster eggs (Gianaroli et al., 1994; Dale et al., 1996).

#### Voltage-gated potassium channels (K_V_)

In mouse eggs, a large conductance voltage-activated K^+^ current was reported in unfertilized eggs. This current is not modulated by cytosolic Ca^2+^ and its activity is linked to the cell cycle, being high in M/G1 and low in S/G2 (Day et al., 1993; Table 1).

Endogenous voltage-activated K^+^ currents, sensitive to Barium blockade, have been reported in *Xenopus* oocyte. However, their function and molecular identity remains elusive (Tokimasa and North, 1996).

### Intercellular channels: connexins

Gap junctions are structures composed by intercellular channels which link the cytoplasm of adjacent cells and allow the exchange of metabolites, nutrients and signaling molecules. Gap junctions are aggregates of connexins (six) organized as large channels (connexons) between cells. In order to develop and mature, primordial oocytes need to establish direct cytoplasmic communication with the granulosa cells through gap junctions. In mice, the connexin responsible for the intercellular communication between oocytes and granulosa cells is connexin 37 (Cx37). The disruption of the gene encoding for Cx37 results in female infertility characterized by oocytes that fail to acquire meiotic competence and showed inappropriate formation of the *corpora lutea*. Ultimately, Cx37 KO females showed anovulation (Simon et al., 1997). Connexin 43 (Cx43) mediates interactions between granulosa cells and may be present in a minor fraction (if any) on the surface of oocytes. The absence of Cx43 in mice ovaries cause impaired postnatal folliculogenesis with failure to develop multiple layers of granulosa cells. Ovary-specific deletion of Cx43 gene generated oocytes that were morphologically abnormal and meiotically incompetent, therefore, cannot be fertilized (Ackert et al., 2001; Kidder and Mhawi, 2002). Connexin 26 (Cx26) has been linked to the implantation process, however, in mice, the specific deletion of the gene encoding Cx26 in the uterine epithelium did not shown any obvious impairment of implantation (Winterhager and Kidder, 2015). In human cumulus cells, the addition of endothelin-1 has been shown to downregulate Cx26, blocking the resumption of meiosis and promoting the germinal-vesicle stage (Cui et al., 2018).

## Conclusions

From the brief overview of the regulation and function of ionic conductances during fertilization, the egg-to-embryo transition and early embryogenesis, it is clear that channels play a fundamental role in mediating these processes. In *Xenopus* oocytes, the regulation of various ion channels at the PM and the ER membrane have been well characterized and their relative contribution to fertilization are fairly well defined. In contrast, there remains much to be learned about mammalian systems. For example, here, we have revisited the channels responsible for the electrical blockade to polyspermy, well-characterized in *Xenopus* but controversial in mammalian oocytes. Currently, the scientific evidence shows that for mammalian eggs, the blockade of polyspermy must be a combination of mechanisms, including changes in the membrane potential, reorganization of proteins expressed at the PM and even intracellular re-arrangements (Bianchi and Wright, 2016).

Current evidence collectively argue that Ca^2+^ influx is critical to maintain Ca^2+^ oscillations which are required for egg activation (Kline and Kline, 1992). However, the molecular identity of the channel(s) supporting Ca^2+^ influx during oocyte maturation and fertilization remains a puzzle that needs to be solved. In this regard, it is interesting to notice that current knowledge argue against a fundamental role for TRP channels during oocyte maturation and fertilization in *Xenopus* oocyte (see Table 1). In contrast, in mammals, members of the TRP channel family have been suggested to mediate Ca^2+^ influx during egg-to-embryo transition. Four main channels have been proposed to support Ca^2+^ influx in the egg: ORAI1, TRPV3, Ca_v_ and TRPM7-like channels. Results obtained using genetically modified animals, argue against an essential role for ORAI, Ca_v_ and TRPV3 at fertilization. Assessment of the role of a TRPM7-like channel awaits the generation of an oocyte-specific KO for TRPM7. Additionally, the possibility of a redundant system needs to be addressed. Orchestrated functioning of channels to promote Ca^2+^ influx might be the way to assure egg activation. Fertilization is an essential process in the evolution and maintenance of any sexually reproducing species. Therefore, it is likely that mammals have evolved multiple redundant mechanisms to assure Ca^2+^ influx at fertilization. These need to be sufficient to refill the stores and maintain the Ca^2+^ oscillations for extended periods of time. Current evidence argues that the egg is agnostic regarding the specific molecular pathway that mediates Ca^2+^ influx as long as it is able to maintain Ca^2+^ oscillations. It should be noted, however, that this Ca^2+^ influx needs to be balanced since overexpression of channels mediating Ca^2+^ influx such as ORAI1 disrupts early embryonic development (Lee et al., 2013). In this regard, the generation of genetically modified animals where a combination of channels are KO would be an interesting model to study Ca^2+^ signaling in oocyte physiology.

Ca^2+^ signals are fundamental to activate eggs at fertilization and to support pre-implantation embryo development. Ca^2+^ also plays a role in completion but not in the initiation of meiosis. Ca^2+^ signaling depends on Ca^2+^ influx and Ca^2+^ release from intracellular reservoirs such as the ER. In most cells, including gametes, Ca^2+^ influx and Ca^2+^ release are mediated by ion channels, in addition to other proteins. Figure 1 summarizes the current model for functional Ca^2+^ channels activity at the PM of mouse oocytes, eggs and early embryos. At the GV stage, spontaneous Ca^2+^ oscillations might be controlled by TRPM7-like and Ca_V_ channels (Carvacho et al., 2016). In ovulated eggs, TRPV3 contributes to Ca^2+^ influx (Carvacho et al., 2013). Additionally, Ca_V_ channels have been proposed to play a role in fertilization-triggered Ca^2+^ oscillations. However, the contribution of Ca_v_ channels must be negligible, since no major differences in Ca^2+^ oscillations were observed between *Cacna1h*^−/−^ and *Cacna1h*^−/+^ eggs (Bernhardt et al., 2015). After fertilization, pharmacological blockade of TRPM7-like channels suggest a fundamental role of the protein supporting Ca^2+^ oscillations and pre-implantation development (Carvacho et al., 2016; Bernhardt et al., 2017) (Figure 1). It was suggested that STIM and ORAI might mediate Ca^2+^ influx following store depletion in mouse GV oocytes. Mice lacking *Stim1/2* and *Orai1* did not show any difference in ER Ca^2+^ stores in comparison to WT oocytes (Bernhardt et al., 2017). Nevertheless, the expression of native ORAI proteins has been shown by western blot (Cheon et al., 2013), therefore, ORAI in GV oocytes has been added to the model. Besides the aforementioned channels, we cannot rule out possible contributions of yet unknown channels, thus, this possibility is also indicated (**channel X**, Figure 1). Furthermore, one needs to interpret the KO studies with caution as they do not replicate the normal physiological state despite the fact that currently they provide the best tool available to directly assess the role of such channels. The KO of a channel in the oocyte is likely to remodel the expression and/or activity of other channels and/or transporters during oocyte growth and development using a feedback mechanism to ensure appropriate ionic and cellular homeostasis. As such phenotypes observed from mouse KO studies may not reflect the normal physiological state, despite the fact that they would conclusively define the absolute requirement for a particular gene. In that context, current data collectively argue that Ca^2+^ influx at fertilization in mammals is not mediated by rather multiple redundant pathways.

Studying ion channels in gametes remains technically challenging compared to somatic cells. Due to specialized biology of the gametes, researchers face difficulties in manipulating expression and controlling maternal effects. Different model systems are more amenable to certain experimental approaches than others. For example, given the large size of the oocyte, the *Xenopus* system is ideally suited for expression, imaging and biochemical analysis. However, these advantages create a challenge to visualize changes that occur deep in the oocyte. In that context, mammalian oocytes are more advantageous but are of limited use for detailed biochemical analysis. Therefore, a comparative approach building on knowledge from different systems is useful, as long as one remain cognizant of the need of individual species to evolve distinct mechanisms to maintain reproductive isolation.

## Author contributions

IC and KM wrote the first draft of the manuscript. MP designed the figure. IC, MP, TM, and KM critically revised the manuscript. IC and KM prepared the manuscript for submission. IC, MP, TM, and KM approved the final version to be published.

### Conflict of interest statement

The authors declare that the research was conducted in the absence of any commercial or financial relationships that could be construed as a potential conflict of interest.
